# Latent KSHV Infection of Endothelial Cells Induces Integrin Beta3 to Activate Angiogenic Phenotypes

**DOI:** 10.1371/journal.ppat.1002424

**Published:** 2011-12-08

**Authors:** Terri A. DiMaio, Kimberley D. Gutierrez, Michael Lagunoff

**Affiliations:** Department of Microbiology, University of Washington, Seattle, Washington, United States of America; University of North Carolina at Chapel Hill, United States of America

## Abstract

Kaposi's Sarcoma (KS), the most common tumor of AIDS patients, is a highly vascularized tumor supporting large amounts of angiogenesis. The main cell type of KS tumors is the spindle cell, a cell of endothelial origin, the primary cell type involved in angiogenesis. Kaposi's Sarcoma-associated herpesvirus (KSHV) is the etiologic agent of KS and is likely involved in both tumor formation and the induction of angiogenesis. Integrins, and specifically integrin αVβ3, have known roles in both tumor induction and angiogenesis. αVβ3 is also important for KSHV infection as it has been shown to be involved in KSHV entry into cells. We found that during latent infection of endothelial cells KSHV induces the expression of integrin β3 leading to increased surface levels of αVβ3. Signaling molecules downstream of integrins, including FAK and Src, are activated during viral latency. Integrin activation by KSHV is necessary for the KSHV-associated upregulation of a number of angiogenic phenotypes during latent infection including adhesion and motility. Additionally, KSHV-infected cells become more reliant on αVβ3 for capillary like formation in three dimensional culture. KSHV induction of integrin β3, leading to induction of angiogenic and cancer cell phenotypes during latency, is likely to be important for KS tumor formation and potentially provides a novel target for treating KS tumors.

## Author Summary

Kaposi's Sarcoma (KS) is the most common tumor of AIDS patients world-wide and is characterized by very high vascularization. The main KS tumor cell type is the spindle cell, a cell of endothelial origin. Kaposi's Sarcoma-associated herpesvirus (KSHV), the etiologic agent of KS, is found predominantly in the latent state in spindle cells. In this study we examined how KSHV alters endothelial cells to induce phenotypes common to angiogenesis and tumor formation. Integrins are cell surface adhesion and signaling proteins that can be involved in tumor growth and tumor angiogenesis. We found that KSHV infection of endothelial cells leads to increased expression of integrin β3, a molecule that, when paired with its cognate α subunit, αV, has been shown to be critical for tumor-associated angiogenesis. KSHV infection promotes angiogenic phenotypes in endothelial cells including adhesion, motility and capillary morphogenesis, and these phenotypes require expression and signaling through integrin β3. Therefore, KSHV induction of integrin beta3 and downstream signaling is required for the induction of phenotypes thought to be critical for KS tumor formation. αVβ3 inhibitors are in clinical trials for inhibition of tumors and we propose that these inhibitors may be clinically relevant for treatment of KS tumors.

## Introduction

Kaposi's sarcoma-associated herpesvirus (KSHV), a gamma herpesvirus, is the etiological agent for Kaposi's sarcoma (KS). KS is the most common tumor in AIDS patients world-wide, and is the most commonly reported tumor in parts of central Africa [1], [2]. KS tumors are highly vascularized, with abnormal, leaky vasculature, and excess inflammation and edema. The histopathology of KS tumors supports a role for angiogenesis in tumor formation. The primary cell type of KS lesions are spindle-shaped endothelium-derived cells aptly named spindle cells. Nearly all spindle cells support latent KSHV infection, although a low percentage of cells undergoing lytic reactivation are always present [3].

KSHV can infect many types of cells in culture including endothelial cells [4], [5]. KSHV infection of endothelial cells in culture leads to predominantly latent infection with a similar low percentage of cells undergoing lytic replication as in the KS tumor [4], [6]. KSHV infection of endothelial cells can promote angiogenesis related phenotypes, including increased stability of tubules formed by macrovascular endothelial cells, induction of angiogenesis and capillary morphogenesis in low growth factor conditions, and enhanced migration and invasion [7]–[11]. Furthermore, KSHV infection can induce increased expression and secretion of signaling factors involved in angiogenesis, such as vascular endothelial growth factor (VEGF). Both VEGF-A and –C are expressed by KSHV-infected endothelial cells [12], [13]. Interestingly, KSHV infection promotes the upregulation of both VEGF receptor 1, a blood vasculature marker, and VEGF receptor 3, a marker for lymphatic endothelium [13]–[17]. The upregulation of both VEGF receptors suggests KSHV-infected cells are more sensitive to the growth and migratory effects of VEGF than the surrounding uninfected endothelium. KSHV infection also leads to upregulation of other molecules with important roles in the regulation of angiogenesis. KSHV-induced expression of cyclooxygenase-2 (COX-2) as well as angiogenin was shown to be important for the maintenance of latency, as well as inflammatory cytokine expression and capillary morphogenesis [18]. KSHV infection of endothelial cells upregulates several members of the angiopoietin family of growth factors, including angiopoietin-2 and angiopoietin-like 4, which are involved in regulating angiogenic remodeling and vessel stabilization [19]-[21]. In addition to secretion of growth factors, KSHV infection promotes disruption of adherens junctions, allowing for increased vascular permeability and invasion [22]–[25]. Furthermore, there have been several studies examining the role of other molecules and signaling pathways which have been implicated in KSHV-induced angiogenesis [26]–[34].

Several KSHV genes have been shown to regulate expression of genes involved in angiogenesis. For example, both vIRF3 and the glycoprotein K1 promote VEGF expression [35], [36]. Other genes were found to have angiogenic chemoattractant properties, such as the viral homologs to macrophage inflammatory proteins (vMIPs I–III; [37], [38]). The viral G-protein coupled receptor (vGPCR) is a constitutively active signaling receptor that has been linked to a variety of angiogenic signaling pathways [21], [39]–[44]. However, vGPCR and the other genes mentioned are primarily expressed during lytic replication and they have not been shown to be necessary for the induction of angiogenic phenotypes in the context of viral infection. Since only a small percentage of the infected cells undergo lytic replication, it is unknown if these proteins are sufficient to promote an angiogenic phenotype in the more abundant latently infected endothelial cells.

During latency in endothelial cells, only five viral genes are expressed, including the latency-associated nuclear antigen (LANA-1). LANA-1 has been shown to play a role in a host of processes including maintenance of the viral genome and host cell survival (reviewed in [45]). In addition to these functions, LANA-1 may play several roles in promoting angiogenesis. Expression of LANA-1 in endothelial cells induces upregulation of angiogenin, which may aid in induction of angiogenesis by VEGF and basic fibroblast growth factor [10]. Furthermore, LANA-1 interaction with Daxx reduces its repression on Ets-1-dependent VEGF receptor expression [46]. While it is apparent that KSHV genes are capable of inducing angiogenic pathways, a better understanding of how viral infection alters the host cell to induce angiogenesis is still needed.

Angiogenesis is a complex process that is tightly regulated by a delicate balance of pro- and anti-angiogenic factors. However, many pathogenic processes, such as tumor formation, shift this balance to promote continual vascular growth. How the different angiogenic signaling factors interact and are regulated is only partly understood. Along with secreted cytokines and growth factors, other signaling proteins such as integrins have been shown to regulate endothelial cell activation and angiogenesis. Integrins are cell surface proteins that link the extracellular matrix to the cytoskeleton. They form dimers of α and β subunits that recognize extracellular matrix (ECM) proteins and, upon ligand binding, undergo conformational changes and recruit intracellular adaptor and signaling molecules. Integrins can induce activation of focal adhesion kinase (FAK) leading to activation of Src kinase. Integrin signaling complexes play a role in a number of cellular processes such as adhesion to the ECM, migration, and cell survival during suspension, all of which are essential functions for endothelial cells during the process of angiogenesis (reviewed in [47]–[49]). In particular, αVβ3 integrin has been shown to be upregulated in the vasculature associated with a number of tumor types and has been linked to regulation of angiogenesis [50]–[52]. Antagonists of integrin αVβ3 inhibit tumor angiogenesis and tumor growth in a variety of animal models of cancer [52]–[55]. Interestingly, αVβ3 integrin is a receptor for several viruses, including KSHV [56]. KSHV glycoprotein B (gB) can associate with integrins on the cell surface leading to increased signaling, adhesion, and to cytoskeletal rearrangements. However, previous studies have not examined the role of integrins during latent KSHV infection.

We have identified a role for integrin β3 during latent infection of KSHV in endothelial cells. Infected cells upregulate integrin β3 expression leading to increased cell surface αVβ3. Latently infected endothelial cells become more adherent to integrin ligands fibronectin and vitronectin, and are also more migratory than mock-infected cells. These induced phenotypes require RGD-binding integrins, specifically integrin β3. Interestingly, although both uninfected and infected cells organize in three-dimensional culture, infected cells are more sensitive to inhibitors of integrin β3 and its downstream signaling molecules, such as Src kinase. This suggests that during latent KSHV infection there is a shift in endothelial cell signaling that results in a more angiogenic phenotype dependent on αVβ3.

## Results

### Latent KSHV infection upregulates integrin β3 expression

**Figure 1:**
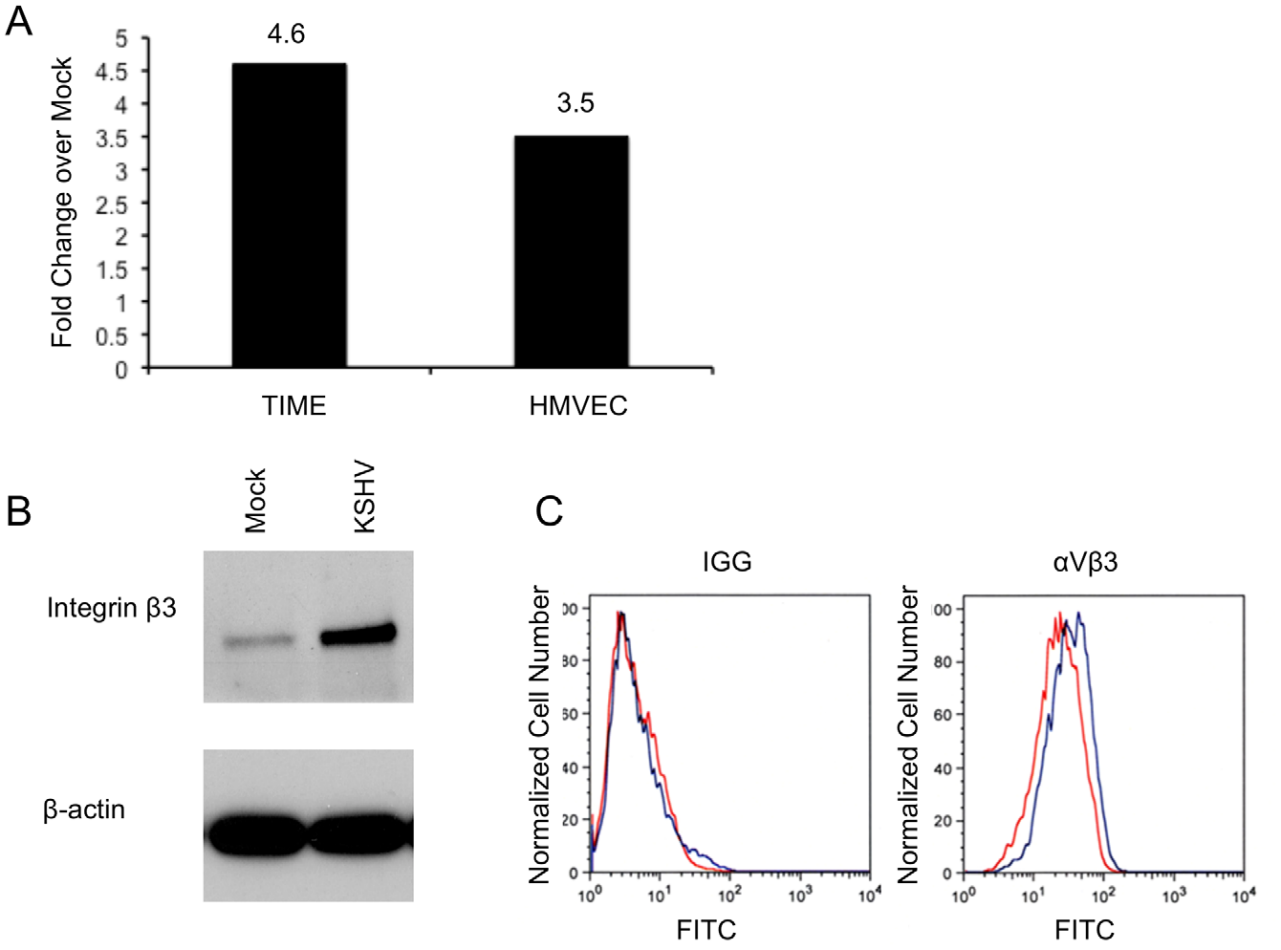
KSHV latent infection increases integrin β3 expression. A) TIME cells or primary human dermal microvascular cells were either mock- or KSHV-infected, and allowed to establish latency for 48 hours. Relative abundances of integrin β3 mRNA were analyzed by one-step real-time RT-PCR. B) 48 hours post-infection mock- or KSHV-infected TIME cells were harvested and cell lysates were separated by SDS-PAGE and stained with an antibody to integrin β3. C) Mock- (red line) or KSHV-infected (blue line) TIME cells were stained with antibody to mouse IGG or αVβ3 and analyzed by flow cytometry.

Our microarray analysis of mock- and KSHV-infected endothelial cells indicated that integrin β3 was significantly upregulated in all our KSHV-infected samples (data not shown). To confirm this we utilized quantitative real-time RT-PCR to measure integrin β3 mRNA levels during KSHV latent infection of endothelial cells. TIME or primary human dermal microvascular endothelial cells were mock- or KSHV-infected and allowed to establish latent infection for 48 hours. RNA from these cells was extracted and subjected to real-time RT-PCR using primers specific to integrin β3. Figure 1A shows that KSHV-infected TIME cells had a 4.6-fold increase in integrin β3 expression as compared to their mock counterparts. In primary endothelial cells (ECs), KSHV induced a 3.5-fold increase in integrin β3 expression over mock infection. In the cultures analyzed, greater than 90% of the infected cells expressed LANA and less than 1% of the cells expressed ORF 59, a marker of lytic infection, indicating that the increase is likely to be a result of latent infection.

We next examined the expression of integrin β3 at the protein level, using western blot analysis and flow cytometry. In TIME cells latently infected with KSHV, total cellular integrin β3 was significantly increased as visualized by Western blot analysis with an antibody specific to integrin β3 (Figure 1B). In endothelial cells αV is the only integrin α subunit that dimerizes with the β3 subunit. Flow cytometric analysis showed a specific increase in cell surface expression of αVβ3 during latent KSHV infection of TIME cells (Figure 1C). The mean fluorescence intensity of mock-infected cells was approximately 22.3, while KSHV infection increased the mean to 31.9. Although there is only a modest increase in cell surface αVβ3 protein expression as compared to total cellular expression, this shift was highly reproducible upon multiple infections with different stocks of virus. Importantly, expression of αVβ3 increased in the majority of cells, indicating that the increased surface protein expression was a property of latently infected cells, not only the low percentage of lytically infected cells.

### Integrin signaling through FAK and Src

**Figure 2:**
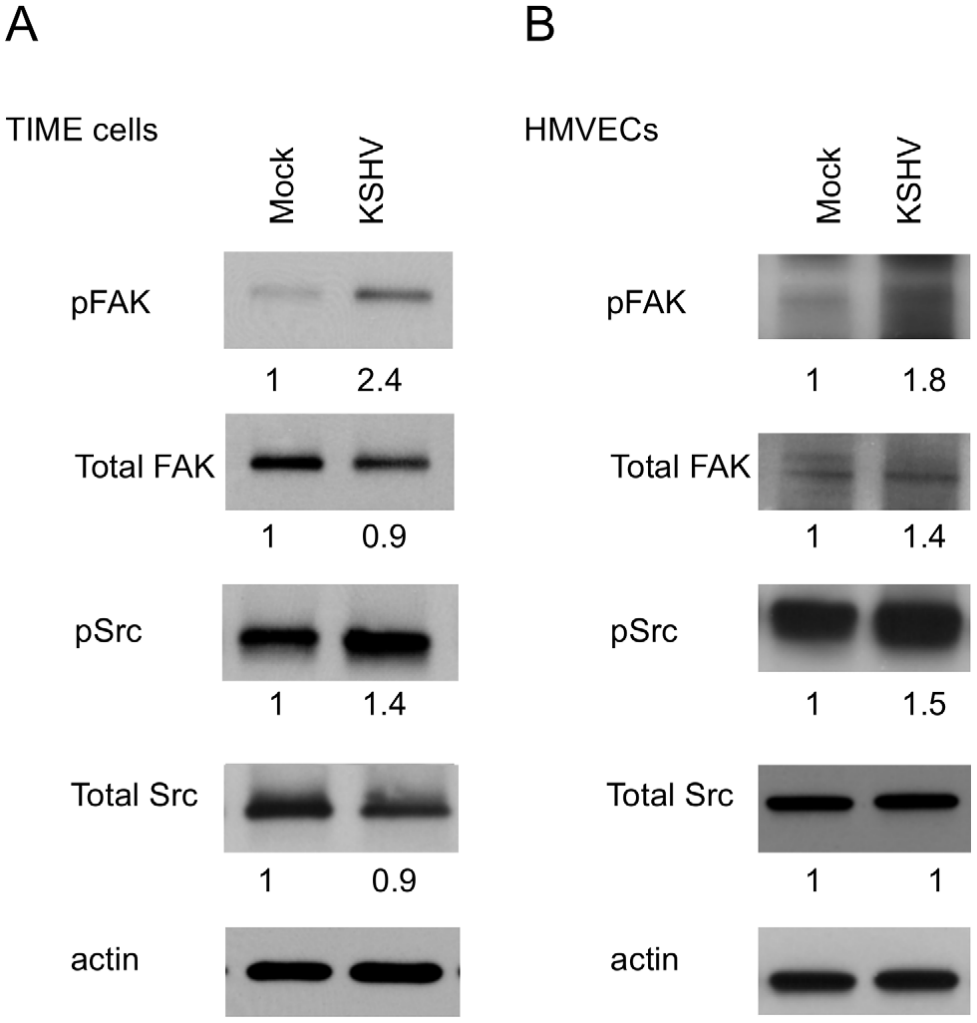
FAK and Src are activated during KSHV latency in endothelial cells. TIME cells (A) or primary dHMVECs (B) were mock- or KSHV-infected. At 48 hpi, cell extracts were analyzed by immunoblot analysis with the indicated phospho-specific or total-protein antibodies. Blots were stripped and probed with an antibody to β-actin as a loading control. The numbers below the lanes indicate the relative abundance of each major band. These experiments were repeated three times with similar results.

Activation of integrins leads to recruitment of the intracellular protein focal adhesion kinase (FAK) and autophosphorylation of FAK on tyrosine 397 [47]. This autophosphorylation provides a binding site for src family kinases, which subsequently become activated. To determine if KSHV promotes integrin signaling through FAK and Src, we analyzed the phosphorylation state of these proteins at 48 hours post infection, after the establishment of latency. Figure 2A shows an increase in FAK phosphorylation on tyrosine 397 in KSHV-infected cells, compared to mock-infected cells. Furthermore, Src is also phosphorylated more heavily on tyrosine 416 in KSHV-infected cells. These experiments were also performed in primary HMVECs, which upon infection undergo a small but reproducible increase in Fak and Src activation (Figure 2B). These data suggest that KSHV latent infection promotes signaling through FAK and Src.

### KSHV promotes focal adhesion complex turnover

**Figure 3:**
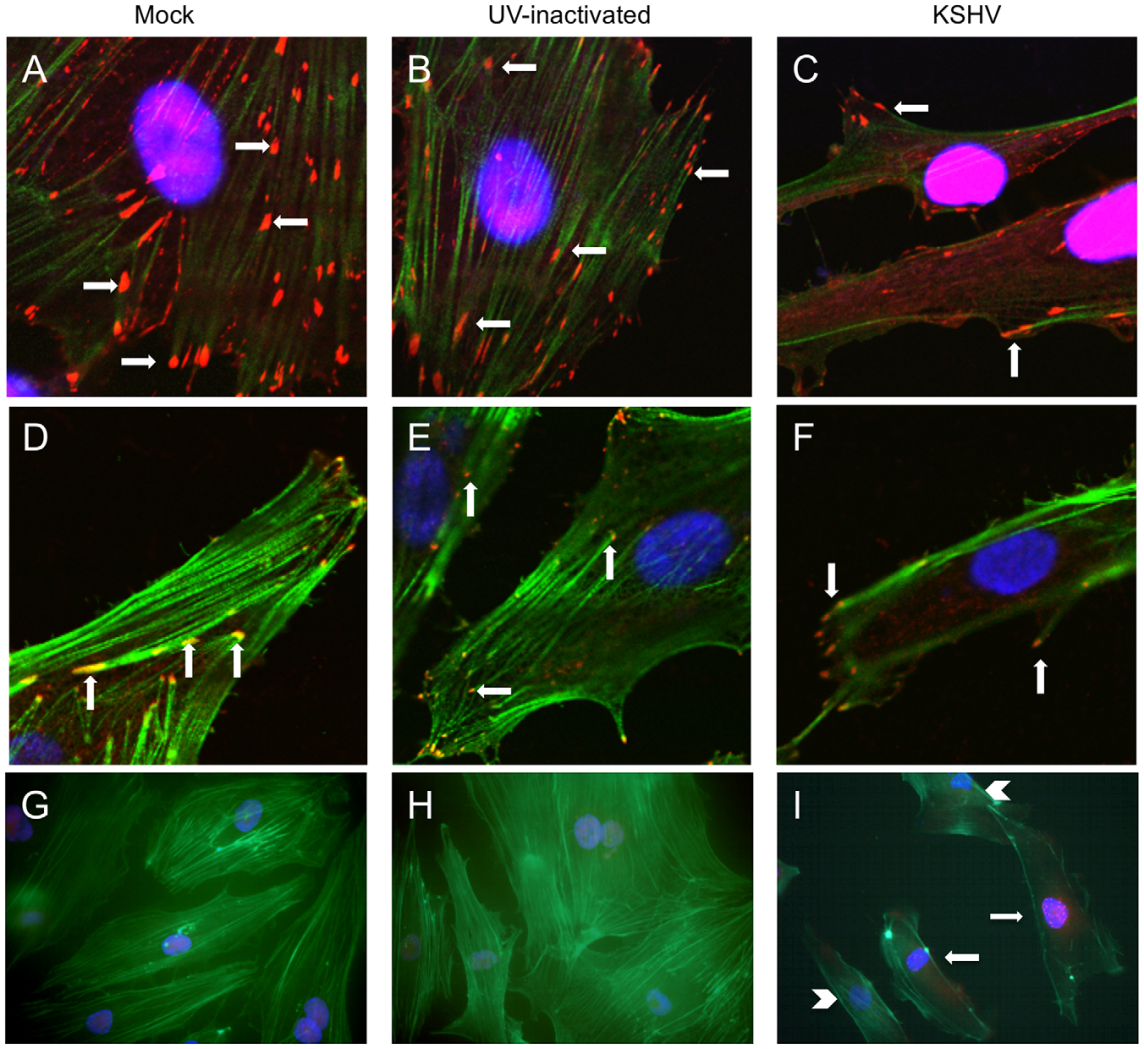
KSHV latent infection promotes turnover of focal adhesions and relocalization of integrin β3. Primary HMVECs were infected with mock (A,D,G), UV-inactivated KSHV (B,E,H), or KSHV (C,F,I) and allowed to establish latency for 48 hours. A–C) Cells were then fixed and stained for vinculin (red) and phalloidin to visualize polymerized actin (green). Cells were mounted in medium containing DAPI (blue). Arrows indicate focal adhesions. Images were taken at 630x magnification. D–F) Cells were stained with antibody to integrin β3 (red) and phalloidin (green). Arrows indicate integrin β3 localized to focal adhesions. Images were taken at 630x magnification. G–I) Cells were stained with antibody to LANA (red) and phallodin (green) as above. Images were taken at 400x magnification. I) Arrows show LANA-positive KSHV-infected cells, while arrow heads point to LANA-negative uninfected cells in the same culture.

A key process during cell migration is the regulation of focal adhesion complexes, groups of signaling molecules that mediate integrin signaling to the actin cytoskeleton. In order to better understand the effect of KSHV induction of integrin β3 on endothelial cell morphology, we examined the formation of focal adhesion complexes in mock- or KSHV-infected cells. Figure 3A shows the localization of the focal adhesion component vinculin in mock-infected cells, which have many centrally localized focal adhesions. The cells were also stained with phalloidin to identify polymerized actin which is present throughout the cells. In contrast, at 48 hours post-infection, endothelial cells latently infected with KSHV have fewer focal adhesions but they are strongly localized to the periphery, suggesting changes in the formation and turnover of the focal adhesions (Figure 3C). The polymerized actin also localizes to the periphery of the cell during latent infection. The fewer focal adhesions and their localization at the periphery suggest higher turnover and increased migration in KSHV-infected cells.

To eliminate the potential effects of virus binding and entry, UV-inactivated virus was used to infect cells. UV-irradiated virus can bind and enter cells but there is no viral gene expression, as determined by immunofluorescence with anti-LANA antibodies. UV-inactivated virus did not alter the localization of focal adhesions at 48 hours post-infection (Figure 3B). Interestingly, in a mixed population of infected and uninfected cells, only infected cells (as determined by staining with LANA-1 antibody) had peripheral localization of focal adhesions. Uninfected cells in the same population had more centrally localized focal adhesions, similar to mock-infected cells (data not shown).

The cells were similarly stained with phalloidin and antibodies to integrin β3. Integrins are known components of focal adhesion complexes and have previously been shown to localize with vinculin in these complexes [47], [48], [57]. As expected, integrin β3 had a very similar pattern of relocalization to the periphery as was seen with antibodies to vinculin (Figure 3D–F). These data suggest that latent KSHV infection promotes formation and turnover of focal adhesions and that integrin αVβ3 is activated and localized to the newly formed focal adhesions. These observations are visible throughout the culture, as can be seen in Figure 3G–I, where multiple cells are shown stained for the latent antigen LANA (red) and the actin cytoskeleton (green). Latently infected cells (Figure 3I, arrows) show peripheral organization of actin filaments, compared to the neighboring uninfected cells in the same culture (Figure 3I, arrow heads), which have actin organization similar to mock-infected or UV-inactivated virus (Figure 3G–H). Furthermore, peripheral focal adhesions can be detected in greater than 90% of the highly infected cells. In contrast, ORF59 is detected in less than 1% of the cells (data not shown). In our endothelial infections the KSHV late lytic protein K8.1, is detected in an even lower percentage of the cells than ORF59 and we could only detect gB in a very low percentage of cells as well (data not shown). Importantly, gB has previously been shown to be detected only in the low percentage of cells undergoing lytic replication [58]. Together, these data indicate that the vast majority of cells with peripheral focal adhesions are latently infected and, while lytic replication could play some paracrine role, it is unlikely to play a primary role in organization of focal adhesion complexes during KSHV infection as uninfected cells would be altered as well in this scenario.

### KSHV promotes endothelial cell adhesion and migration in an RGD- and integrin β3-dependent fashion

**Figure 4:**
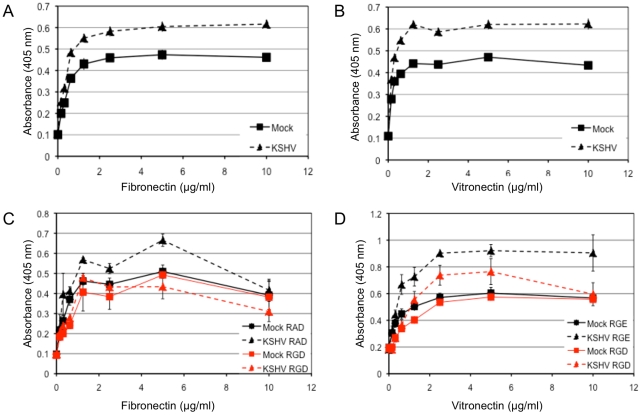
KSHV latent infection promotes adhesion to fibronectin and vitronectin in an integrin-dependent fashion. TIME cells were mock- or KSHV-infected and 48 hours post-infection cells were plated onto 96-well plates coated with fibronectin (A) or vitronectin (B). Cells were allowed to adhere for 90 minutes after which non-adherent cells were washed off. The number of adherent cells was determined by alkaline phosphatase activity. Mock-infected cell adhesion is indicated with squares and KSHV-infected cell adhesion is indicated with triangles. C and D) 48 hours post-infection, mock- (squares) and KSHV- (triangles) infected cells were pretreated with RGD-containing (red lines) or control peptide (RAD for (C) and RGE for (D), black lines) for 15 minutes on ice before assaying for adhesion to fibronectin (C) or vitronectin (D).

We next wanted to determine whether KSHV latent infection promotes an angiogenic phenotype in endothelial cells through the activation of αVβ3. Adhesion and migration of endothelial cells are both critical for angiogenesis. Figure 4 shows the adhesion of mock- or KSHV-infected cells to various concentrations of the extracellular matrix proteins fibronectin and vitronectin, both of which contain the arginine-glycine-aspartic acid (RGD) motif that is recognized by a subset of integrins, including αVβ3. Latent infection by KSHV enhanced the adhesion of endothelial cells to both fibronectin (Figure 4A) and vitronectin (Figure 4B) compared to mock-infected cells. None of the cells adhered to either collagen I, which lacks an RGD motif, or laminin, suggesting the increased adhesion is specific to fibronectin and vitronectin (data not shown). Short RGD-containing peptides will compete with vitronectin and fibronectin binding to RGD-dependent integrins, like αVβ3, and inhibit integrin-dependent adherence to these substrates. Interestingly, an RGD-containing short peptide, but not a control RAD- or RGE-containing peptide, inhibited KSHV-induced adhesion to fibronectin or vitronectin. The same peptides had little effect on mock-infected cells (Figure 4C–D). This indicates that KSHV-infected cells require RGD-binding integrins for increased adherence while mock-infected cells are relatively unaffected by the concentrations of RGD peptides used.

**Figure 5:**
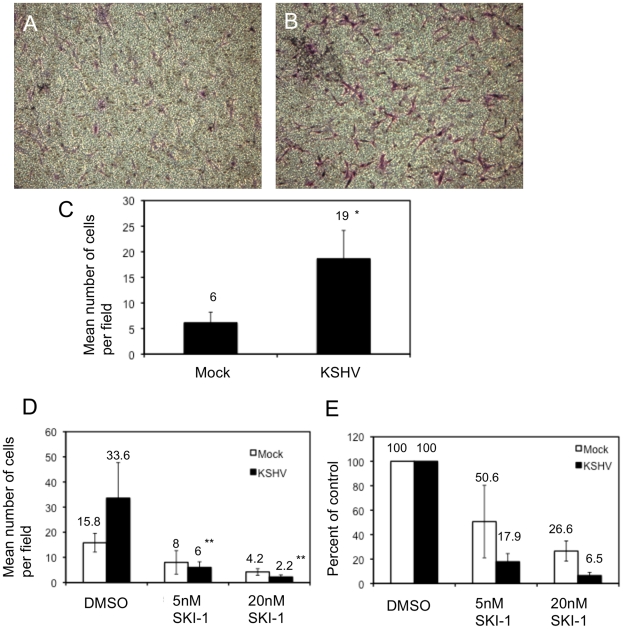
KSHV latent infection promotes endothelial cell transwell migration in a Src-dependent fashion. Cells were infected with mock or KSHV and, 48 hours post-infection, were plated on fibronectin-coated transwells. Cells were allowed to migrate through the pores at 37°C for 1.5 hours and subsequently fixed and stained with crystal violet. The cells on the upper side of the membrane were removed with a cotton swab, the membranes were mounted on slides and the cells that migrated were photographed. A and B) Representative images of migrated mock- (A) and KSHV-infected (B) cells. C) Quantification of a representative experiment showing the number of migrated cells per field for 10 fields counted per transwell. Asterisk indicates a *p*-value of less than 0.05 compared to mock-infected cells. D–E) Cells (48 hpi) were treated with the Src kinase inhibitor SKI-1 at the time of plating on fibronectin-coated transwells and are presented as number of cells per field (D) and percent inhibition by SKI-1 (E). Double asterisks indicate a *p*-value of less than 0.005 compared to DMSO control.

In addition to adhesion, integrin activation can promote migration of endothelial cells through formation and turnover of focal adhesions. In Figure 3 we demonstrated a strong reorganization of focal adhesions during KSHV latency in endothelial cells. We therefore examined the effect of KSHV infection on endothelial cell migration. 48 hours post-infection, endothelial cells were seeded on a transwell membrane with 8 µm pore size and allowed to migrate for 1.5 hours. Approximately three times more KSHV-infected endothelial cells migrated through the pores as compared to mock-infected cells (Figure 5A–C). This increase in migration was not due to increased proliferation, since KSHV infection does not promote proliferation in TIME cells as determined by uptake of BrdU (data not shown). To determine whether this enhanced migration was due to increased integrin signaling, we used a Src kinase inhibitor to block downstream integrin signaling. The Src kinase inhibitor led to a decrease in the mean number of mock- and KSHV-infected cells that migrated through the pores (Figure 5D). However, the lower dose of the Src kinase inhibitor decreased the migration of mock-infected cells by approximately 50% while migration of KSHV-infected cells was reduced more than 80% (Figure 5E). Therefore, KSHV-infected cell migration is sensitive to Src inhibitors to a greater degree than migration of mock-infected cells.

### Integrin β3 activation is required for KSHV-induced capillary morphogenesis

**Figure 6:**
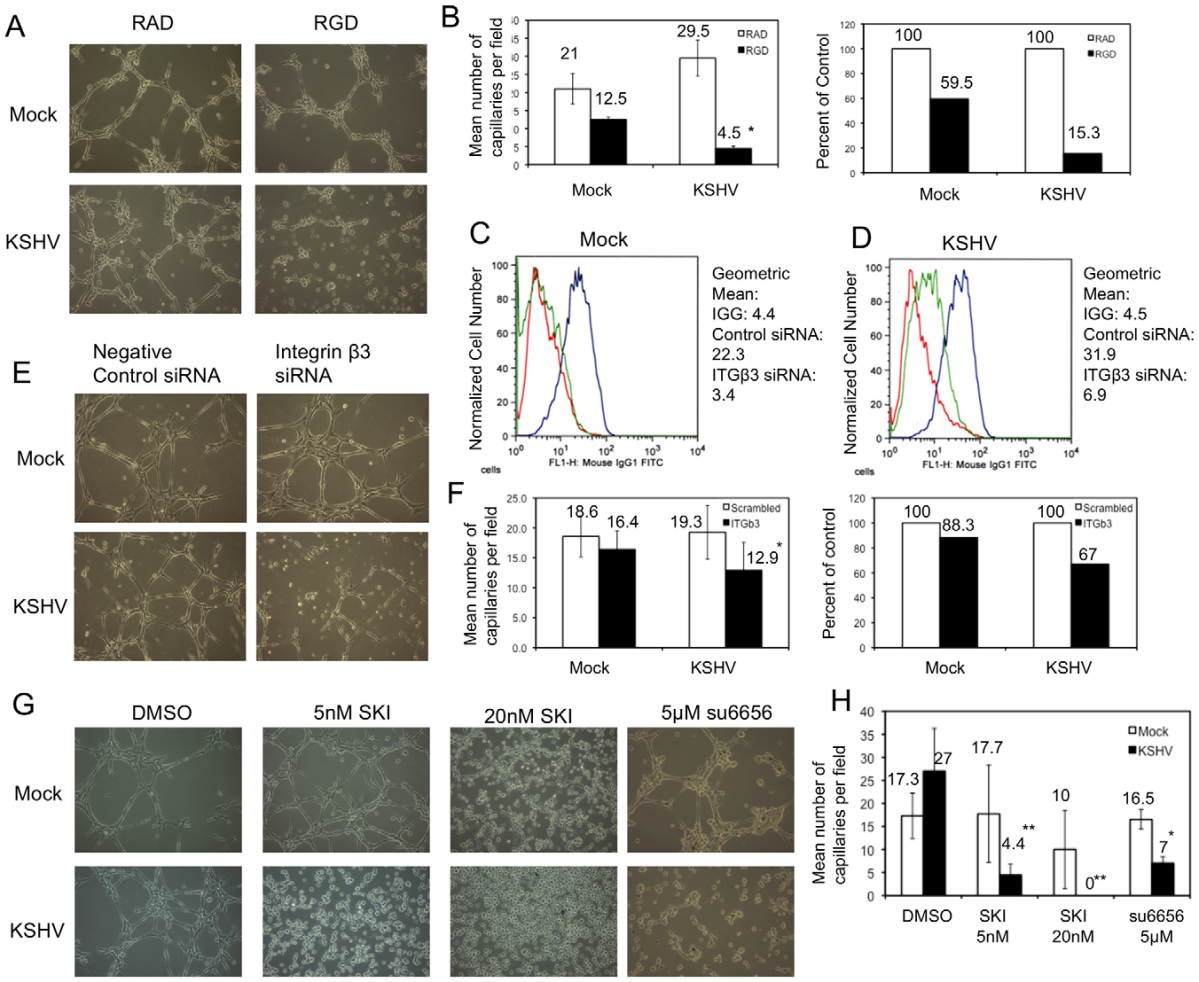
Capillary morphogenesis of KSHV-infected cells requires αVβ3 integrin. A)TIME cells were mock- or KSHV-infected and, 48 hpi, plated on Matrigel in the presence of either RAD- or RGD-containing peptide in complete medium. B) The data in the graphs are the mean number of capillaries per field (left) and the percent of RAD peptide controls (right). C–D) TIME cells were mock- (C) or KSHV-infected (D), and, at 24 hpi, transfected with either control siRNA or siRNA specific to integrin β3. Cells were stained with antibody to mouse IGG (red line) or αVβ3 integrin (blue line: control siRNA; green line: integrin β3 siRNA) and analyzed by flow cytometry. E) At 72 hours post infection and 48 hours post transfection of integrin β3 siRNA, mock- and KSHV-infected cells were plated on Matrigel in complete media and cultured for 6 hours. F) Quantitation of three separate experiments similar to the one shown in E. G) TIME cells were mock- or KSHV-infected and, 48 hpi, plated on Matrigel in the presence of either DMSO, SKI-1, or su6656. H) Quantification of three separate experiments similar to that shown in G. Asterisks indicate a *p*-value of less than 0.05 compared to DMSO or negative control. Double asterisks indicate a *p*-value of less than 0.005 compared to DMSO control.

KSHV latent infection leads to small increases in the ability of endothelial cells to organize into capillary-like structures in three-dimensional culture at 6 hours after seeding. However, these effects are not consistently statistically significant (our unpublished observations and Figure 6A). Due to the increased integrin activity and signaling, we hypothesized that KSHV infection may promote capillary morphogenesis through a different pathway than uninfected cells. Therefore, we examined the role of integrin β3 in the ability of KSHV-infected cells to organize in three-dimensional culture on Matrigel. We first tested whether RGD-binding integrins were involved by using an RGD-containing peptide to block integrin activity. Interestingly, in the presence of the RGD-containing peptide, KSHV-infected cells formed approximately 85% fewer capillary-like structures while mock-infected cells lost only 40% of their activity (Figure 6A and B). This indicates that KSHV-infected cells are more sensitive to inhibition of integrin activation as compared to mock-infected cells and suggests that KSHV-infected cells become reliant on integrin activity for this angiogenic phenotype.

In order to determine if this effect was due specifically to integrin β3 we used siRNA to knock down integrin β3 protein expression. Cells were transfected with siRNA 24 hours post-infection with KSHV and harvested for analysis 48 hours later (at 72 hours post-infection). Figure 6 (C and D) shows that the expression of αVβ3 integrin complexes is significantly reduced upon transfection of integrin β3 siRNA. Interestingly, αVβ3 expression is reduced more strongly in mock-infected cells (Figure 6C) than in KSHV-infected cells (Figure 6D). Mock-infected cells with control siRNA have a geometric mean fluorescence of 22.3, which drops to 3.4 in cells expressing integrin β3 siRNA, a greater than 6.5 fold decrease in expression. In contrast, in KSHV-infected cells, the geometric mean fluorescence of integrin β3 goes from 31.9 in control siRNA-expressing cells to 6.9 in β3 siRNA-expressing cells, which is still above background fluorescence levels and is a 4.6 fold decrease. This is likely due to the increased integrin β3 transcript levels in KSHV-infected cells. Integrin β3 specific siRNA impaired KSHV-induced capillary morphogenesis (Figure 6E and F, *p*-value = 0.003) but had little effect on mock-infected cells (a 33% versus 12% decrease in capillary formation respectively), despite the small amount of residual integrin β3 expression in KSHV-infected cells with the β3 specific siRNA. This suggests that the KSHV-induced angiogenic phenotype specifically requires integrin β3 expression.

To further confirm that integrin activation and signaling during KSHV latent infection is required for the KSHV-induced angiogenic phenotype, we next determined whether Src kinase activity is required for KSHV-induced capillary morphogenesis. Mock- and KSHV-infected cells were treated with the indicated concentrations of Src kinase inhibitor SKI-1 or DMSO alone and plated on Matrigel matrix. Similar to the effects of inhibition by the RGD-containing peptide or integrin β3 siRNA, inhibition of Src kinase had a greater impact on KSHV-infected cells than on mock-infected cells (Figure 6G and H). KSHV-infected cells treated with 5 nM SKI-1 had an approximate 5-fold decrease in the number of capillaries formed compared to DMSO-treated cells. In contrast, mock-infected cells retained nearly complete ability to organize on Matrigel. At higher concentrations of SKI-1, capillary formation of KSHV-infected endothelial cells was completely eliminated while mock-infected cells had only modest decreases in capillary formation. To further demonstrate that the effects seen were due to inhibition of Src, another Src kinase inhibitor, su6656, was used. Su6656 had a similar effect on KSHV-infected cells as SKI-1 (Figure 6G and H). Similar experiments with primary HMVECS yielded comparable results (data not shown). Thus, three different methods of inhibiting integrin signaling – RGD peptides, siRNA to integrin β3, and chemical inhibition of downstream integrin signaling – blocked capillary formation by KSHV infected endothelial cells in a three dimensional matrix more significantly than in mock infected cells.

## Discussion

During angiogenesis, activated endothelial cells disrupt the extracellular matrix, proliferate and migrate towards angiogenic stimuli, gain traction into new areas through increased adhesion to specific substrates, and organize into preliminary vasculature. These activities are necessary for vascularization of solid tumors and contribute to the promotion of tumor formation and metastasis. As has been described by others, KSHV is likely to promote angiogenesis in a paracrine fashion to promote new blood vessel growth in the KS tumor [10], [12], [19]–[21], [28], [41], [59]. Here we found that KSHV also directly induces angiogenic phenotypes in latently infected cells. KSHV latent infection of endothelial cells increased cell motility and cell adhesion. Therefore, angiogenic phenotypes induced in latently infected endothelial cells may promote angiogenesis in KS tumors but may also directly be involved in the activation of the endothelial tumor cells themselves.

Integrins are involved in many processes of both angiogenesis and oncogenesis. Integrins have been shown to be critical for angiogenic phenotypes such as adhesion, migration, and anchorage-independent survival (reviewed in [47], [48]). Integrins have also been shown to be important in oncogenesis of many cell types (reviewed in [60]). In particular, integrin αVβ3 is expressed on blood vessels in human tumor biopsy samples, but not on vessels in normal human tissues, suggesting a role in tumor-associated angiogenesis [50], [51]. Furthermore, inhibition of integrins, and in particular αVβ3, can inhibit angiogenesis and promote regression of tumors by inducing apoptosis of endothelial cells [52]–[55]. We found that KSHV not only induces the expression of integrin β3 leading to increased surface expression of αVβ3, but latent infection also leads to the activation of αVβ3 and downstream activation of FAK and Src. Importantly, integrin signaling, specifically αVβ3, is necessary for KSHV induction of angiogenic phenotypes including cell motility and adhesion.

Regulation of angiogenesis is a complex process that involves cross talk between multiple signaling pathways. Integrin signaling complexes have been shown to interact with angiogenic growth factor receptors, such as VEGF and basic fibroblast growth factor [48]. Specifically, interaction between αVβ3 and VEGF receptor 2 can lead to phosphorylation of the cytoplasmic tail of β3 as well as increased phosphorylation of VEGF receptor 2 in a Src-dependent fashion [61]. Interestingly, β3 knockout mice exhibit increased pathological angiogenesis, due to increased expression and signaling through VEGF receptor 2 [62], [63]. However, knock-in expression of a mutant β3 that lacks phosphorylation sites blocks VEGF-induced pathological angiogenesis in these mice [64]. Integrin αVβ3 also plays a role in regulating the expression of VEGF; its activation can increase VEGF secretion by tumor cells [65]. Thus, upregulation of VEGF and its receptors by KSHV likely acts in concert with increased αVβ3 signaling to promote angiogenesis.

Interestingly, KSHV-infected cells become dependent upon αVβ3 for capillary formation. Inhibition of integrin signaling and αVβ3 had small effects on the ability of uninfected endothelial cells to form capillary-like structures on Matrigel. However, during KSHV latency, capillary formation is extremely sensitive to inhibitors of αVβ3 and integrin signaling. In uninfected cells αVβ3 is localized with focal adhesions all over the cell, while in infected cells αVβ3 is localized to the focal adhesions at the periphery. It is possible that the dramatic change in focal adhesions and polymerized actin in the infected cells leads to increased reliance on αVβ3. However, whether the increased sensitivity is due to a switch in the infected cell solely to integrin signaling for capillary formation or if there is a decrease in a compensatory pathway is unknown. In either event, this provides a good target for therapy, as the latently infected cells are more sensitive than mock-infected cells to inhibitors of integrin signaling.

Integrins are also used for KSHV binding and entry into cells. It was first demonstrated that α3β1 was a receptor for KSHV on endothelial cells [66]. Subsequently it was shown that gB binds to αVβ3 and that αVβ3 may be the dominant integrin for binding and entry [56]. Interestingly, both of these integrins together with CD98 may form an entry complex [67]. During binding and entry, gB binding to integrins leads to activation of the integrins and subsequent activation of FAK and focal adhesions [68]–[70]. While this phenomenon is similar to what we describe, we found that activation of FAK and Src also occurs during latency and, at the time points we examined, this is not due to initial binding and entry of the virus. Additionally, gB has previously been shown to only be expressed in cells undergoing lytic replication and cannot be detected at the late times post-infection that we examined [58]. While cells undergoing lytic replication are present in our cultures, less than 1% of the cells express ORF 59, a common marker of lytic infection. Using an immunofluorescence assay we find that most of the infected cells have altered focal adhesions at the periphery. Furthermore, the bulk of the cells shift expression of αVβ3 and the bulk of the population is more motile and more adherent, indicating that the phenomenon we are examining is occurring in latently infected cells. Importantly, in cultures where we have a mixed population of uninfected and KSHV-infected cells, only the cells that have LANA expression, i.e. are latently infected, have relocalized actin to the periphery (Figure 3I) and have focal adhesions at the periphery as well (not shown). Taken together, these data all indicate that there is a factor in the latent cells that is necessary for activation of integrin signaling and induction of the angiogenic phenotypes. While paracrine factors from the low percentage of lytically infected cells could play some role in the angiogenic phenotypes described, a latent factor is still required. The viral gene or genes upregulating integrin β3 and activating αVβ3 and FAK are currently unknown and will be the focus of future studies.

Inhibition of integrins and integrin signaling has been proposed as a target for tumor therapy through inhibition of tumor cell growth itself and through inhibition of neo-angiogenesis. A number of inhibitors of integrin signaling are in clinical trials for tumors that have increased levels of integrins and specifically αVβ3 (reviewed in [49], [60]). Importantly, an inhibitor of αVβ3 is in phase3 trials for inhibition of tumor formation. Based on the studies presented here, we believe that αVβ3 may be a good target for therapy to treat KS tumors. It is difficult to target herpesvirus latency due to the limited viral gene expression. However, through a better understanding of the host cell requirements for latency new therapeutic targets can be identified. Integrin β3 induction and activation by KSHV during latency is critical for a number of phenotypes important for tumor growth and latency, making it an attractive target for therapy.

## Materials and Methods

### Cells

Primary human dermal microvascular endothelial cells (hDMVEC) and TIME cells [71] were maintained as monolayer cultures in EBM-2 medium (Lonza) supplemented with 5% fetal bovine serum, vascular endothelial growth factor, basic fibroblast growth factor, insulin-like growth factor 3, epidermal growth factor, and hydrocortisone (EGM-2 media). BCBL-1 [72] and BJAB cells [73] were maintained in RPMI 1640 medium (Celgro; Mediatech, Inc.) supplemented with 10% fetal bovine serum, penicillin, streptomycin, glutamine, and β-mercaptoethanol.

### Viruses and infection

KSHV inoculum was obtained from BCBL-1 cells (5×10^5^ cells/ml) induced with 20 ng of TPA (12-*O*-tetradecanoylphorbol-13-acetate; Sigma)/ml. After 5 days, cells were pelleted, and the supernatant was run through a 0.45-µm-pore-size filter (Whatman). Virions were pelleted at 30,000xg for 2 h in a JA-14 rotor, Avanti-J-25 centrifuge (Beckman Coulter). The viral pellet was resuspended in EGM-2 without supplements. KSHV infections of TIME and primary hDMVEC were performed in serum-free EBM-2 supplemented with 8 ug/ml polybrene for 3 h, after which the medium was replaced with complete EGM-2. Mock infections were performed identically except that concentrated virus was omitted from the inoculum. For all experiments, infection rates were assessed by immunofluorescence using antibodies against the latency-associated nuclear antigen (LANA) and the lytic protein ORF59. In all infections performed with wild-type (wt) KSHV >85% of the cells were LANA-positive and <1% were ORF59-positive. UV inactivation of KSHV viral stocks (5×1,200 µJ) was performed in a UV Stratalinker 1800 (Stratagene).

### RNA isolation and quantitative RT-PCR

Total RNA was isolated from TIME cells using the RNeasy Plus Minikit (Qiagen). One hundred or 500 ng of total RNA was used in a SuperScript III, Platinum SYBR green, one-step, quantitative reverse transcription PCR (RT-PCR; Invitrogen) according to manufacturer's protocols with the primers for either GAPDH (glyceraldehyde-3-phosphate dehydrogenase) (forward, 5′-AAG GTG AAG GTC GGA GTC AAC G-3′; reverse, 5′-TGG AAG ATG GTG ATG GGA TTT C-3′) or integrin β3 (forward, 5′-GCA AGG ATG CAG TGA ATT GT-3′; reverse, 5′-CTT GGG ACA CTC TGG CTC TT-3′). Relative abundances of integrin β3 mRNA were normalized by the delta threshold cycle method to the abundance of GAPDH, with mock-infected TIME cells set to 1. Error bars reflect standard errors of the means (four experiments).

### Immunoblot analysis

Cells were harvested with a cell scraper and pelleted using the Sorvall RT7 Plus centrifuge at 4°C. An aliquot of the cells was seeded onto chamber slides for immunofluorescence analysis. Cell pellets were washed once in cold phosphate-buffered saline and then resuspended in lysis buffer (20 mM Tris [pH 7.0], 2 mM EGTA, 5 mM EDTA, phosphatase inhibitors, protease inhibitors, and 1% Triton X-100). Samples were sonicated, rocked at 4°C for 30 minutes, and then spun at 6,000 x *g* at 4°C. Cell extracts were fractionated on a sodium dodecyl sulfate-polyacrylamide gel electrophoresis gel, and the proteins were transferred electrophoretically to Immobilon P polyvinylidene difluoride membranes (Millipore) in Tris-glycine buffer (25 mM Tris, 192 mM glycine, 20% methanol). Blots were incubated with the indicated antibody (dilutions: 1∶1000 for anti-integrin β3, 1∶1000 for anti-phosphoFAK and anti-phosphoSrc; 1∶20,000 for anti-actin) and subsequently with horseradish peroxidase-conjugated goat anti-mouse or rabbit immunoglobulin G (1∶10,000; IgG; Jackson ImmunoResearch). Immunoreactive proteins were visualized by chemiluminescence using the Amersham ECL Plus Western blotting detection reagents (GE Healthcare). Differences in band intensity were quantified by densitometric methods.

### Immunofluorescence

Mock- or KSHV-infected TIME cells were seeded on LabTek Permanox four-well chamber slides (Fisher Scientific) and fixed with 4% (wt/vol) paraformaldehyde in phosphate-buffered saline. Immunofluorescence was performed as described previously [4]. Briefly, cells were incubated in Tris-Buffered Saline (20 mM Tris, 150 mM NaCl, pH 7.6; TBS) containing 1% normal goat serum followed by incubation with primary antisera at a dilution of 1∶100 (anti-integrin β3; anti-vinculin) or 1∶1,000 (rabbit or rat anti-LANA; mouse anti-ORF59) diluted in TBS containing 1% BSA overnight. Cells were then incubated with fluor-conjugated secondary antibodies (goat anti-rabbit Alexa Fluor 488, goat anti-mouse Alexa Fluor 594, or goat anti-rat Alexa Fluor 488; Molecular Probes/Invitrogen; Fluorescein-conjugated phalloidin; Sigma) for 2 hours. Cells were mounted in medium containing DAPI (4′,6′-diamidino-2-phenylindole) before being viewed under a Zeiss LSM 510 Meta confocal microscope. Images were analyzed using the Zeiss Zen 2009 LE imaging software.

### Transfection of siRNA

siRNA specific to integrin β3 and negative-control oligonucleotides were designed and synthesized by Ambion (Austin, TX). The following oligonucleotide sequences were used: integrin β3 (Ambion identification [ID] no. 112581; sense, 5′-GCU AAU UCU UUG ACC UGU UdTdT-3′) and negative-control siRNA (sense, 5′-AGU ACU GCU UAC GAU ACG GdTdT-3′). At 24 h post-infection, mock- or KSHV-infected TIME cells were transfected with 3 µg siRNA using Amaxa's Nucleofector kit (Cologne, Germany) according to the manufacturer's protocol. The transfection efficiency for siRNA was approximately 90% when it was assessed with 6-carboxyfluorescein-labeled negative-control siRNA. Transfected cells were harvested for analysis after an additional 2 days of incubation at 37°C.

### Flow cytometric analysis

Monolayers of cells grown in 60-mm tissue culture dishes were washed once with phosphate-buffered saline (PBS) containing 0.04% EDTA and incubated with 2 ml of cell dissociation solution (Sigma, St. Louis, MO) to remove cells from plates. Cells were washed once with DMEM containing 10% FBS and fixed in 4% paraformaldehyde in TBS for 10 minutes on ice. Cells were then pelleted and resuspended in 0.5 ml TBS with 1% goat serum for 20 min on ice. Cells were pelleted and incubated with mouse anti-αVβ3 (2 µg/ml; LM609; Millipore) or mouse IGG control prepared in 0.5 ml of TBS with 1% BSA for 30 min on ice. Following incubation, cells were washed twice with TBS/1% BSA and then incubated with anti-mouse secondary antibody conjugated to Alexafluor 488 (Invitrogen; diluted 1∶200 in 0.5 ml of TBS containing 1% BSA) for 30 min on ice. The stained cells were washed twice with TBS containing 1% BSA, resuspended in 0.5 ml of TBS containing 1% BSA, and analyzed by FACScan caliber flow cytometer (Becton-Dickinson, Franklin Lakes, NJ). Data was analyzed using FloJo flow cytometry analysis software (Tree Star, Inc.; Ashland, OR).

### Cell adhesion assays

Cell adhesion assays were performed using Nunc 96-well Maxisorp plates as described previously [74]. Briefly, wells were coated with different concentrations of collagen, fibronectin, laminin, or vitronectin in TBS containing 2 mM each of CaCl_2_ and MgCl_2_ overnight at 4°C. The next day, plates were washed with TBS and blocked with 200 µl of TBS Ca/Mg containing 1% BSA for 1 hour at room temperature. Cells were resuspended in 20 mM HEPES, 150 mM NaCl, 4 mg/ml BSA (pH 7.4) and plated at 5×10^4^ cells/well. Cells were allowed to adhere at 37°C in a humidified incubator for 1.5 hours. Nonadherent cells were gently washed off with TBS containing Ca/Mg, and the number of adherent cells was determined by measuring the standard intracellular acid phosphatase activity. For inhibition studies on fibronectin, cells were treated with GRADSPK or GRGDSPK peptides (Sigma) in HEPES buffer for 15 minutes on ice prior to plating. For inhibition studies on vitronectin, cells were treated with GRGES or GRGDS peptides (Peptides International) in HEPES buffer for 15 minutes on ice prior to plating.

### Transwell migration

For transwell assays, transwell filters (Costar 3422) were placed in a 24-well dish and coated on the bottom side with 0.5 ml of 2 µg/ml of fibronectin (BD Biosciences) in PBS overnight at 4°C. The filter was rinsed with PBS and then blocked with 0.5 ml of 2% bovine serum albumin (BSA) prepared in PBS for 1 h at room temperature. Following blocking, the filter was rinsed with PBS, 0.5 ml of serum-free DMEM medium was added to the bottom of each well. Cells were resuspended at 1×10^5^ cells/ml in serum free medium and 0.1 ml was added to the top of each well. Each condition was done in duplicate. Following 1.5 hours in a 37°C tissue culture incubator, the cells and medium were aspirated and the upper side of the membrane wiped with a cotton swab. The cells that had migrated through the membrane and attached to the bottom of the filter were fixed with 4% paraformaldehyde and stained with crystal violet. The mean number of cells migrated through the filter was determined by counting ten high power fields (×100). For inhibition studies, cells were incubated with SKI-1 (Calbiochem) for 15 minutes on ice prior to addition to the transwell.

### Three-dimensional culture of endothelial cells

Matrigel (10 mg/ml; BD Biosciences, Bedford, MA) was applied at 0.5 ml/35 mm in a tissue culture dish and incubated at 37°C for at least 30 min to harden. Mock- or KSHV-infected cells were removed using trypsin-EDTA, washed with growth medium once, and resuspended at 1.5×10^5^ cells per ml in growth medium. Cells (1 ml) were gently added to the Matrigel-coated plates, incubated at 37°C, monitored for 6 h, and photographed in digital format using a Nikon microscope. Capillaries were defined as cellular processes connecting two bodies of cells. Ten fields of cells were counted for each condition and the mean and standard deviations were determined. For inhibition studies, GRADSPK or GRGDSPK peptides, SKI-1 (Calbiochem), or su6656 (Sigma) were added at the time of plating on Matrigel.

### Statistics

Statistical differences between groups were evaluated with Student's *t*-test (two-tailed). Mean±SD was shown and a *p*-value of ≤0.05 was considered significant and indicated by asterisk.
